# Verifying the Unique Charge Migration Pathway in Polymeric Homojunctions for Artificial Photosynthesis of Hydrogen Peroxide

**DOI:** 10.1002/advs.202500218

**Published:** 2025-03-05

**Authors:** Qiang Cheng, Jingping Li, Yuxin Huang, Xiufan Liu, Biao Zhou, Qiao Xiong, Kai Wang

**Affiliations:** ^1^ College of Urban and Environmental Sciences Hubei Key Laboratory of Pollutant Analysis and Reuse Technology Hubei Normal University Huangshi 435002 P. R. China

**Keywords:** charge transfer path, hydrogen peroxide synthesis, photocatalysis, polymeric homojunction, s‐scheme mechanism

## Abstract

Artificial photosynthesis for producing high‐value hydrogen peroxide (H_2_O_2_) using carbon nitride‐based systems holds immense potential. However, understanding the charge transfer dynamics in homojunction photocatalysts remains a significant challenge owing to the limitations of current characterization techniques. Here, a polymeric C_3_N_5_/C_3_N_4_ homojunction (CNHJ) is employed as a model system to probe interfacial electron transfer. Bimetallic cocatalysts serve as sensitive probes, enabling in situ tracking of the S‐scheme electron transfer between C_3_N_5_ and C_3_N_4_ via X‐ray photoelectron spectroscopy. Leveraging the unique advantages of this S‐scheme, the CNHJ demonstrates substantially enhanced performance in the two‐electron oxygen reduction reaction, achieving an impressive H_2_O_2_ production rate of 8.78 mmol g^−1^ h^−1^ under visible light irradiation. Furthermore, the system demonstrates robust performance in continuous‐flow setups, under natural sunlight, and in photocatalytic disinfection tests, highlighting its practical potential. This approach offers new insights into dynamic electron transfer mechanisms and paves the way for advancing artificial photosynthesis technologies.

## Graphical abstract

Limited charge separation is a challenge for efficient photocatalysis. This work introduces a polymeric S‐scheme homojunction for unique charge separation and transfer in photocatalytic H_2_O_2_ production.

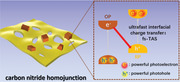

## 1 Introduction

Hydrogen peroxide (H_2_O_2_) is extensively used as an environmentally friendly oxidant and clean liquid fuel, with significant applications across various industrial and medical fields. Notably, it served as a crucial disinfectant during the recent COVID‐19 pandemic.^[^ ^1^, ^2^, ^3^, ^4^, ^5^, ^6^ ^]^ The conventional anthraquinone process for H_2_O_2_ production is energy‐intensive, while direct catalytic methods involving H_2_ and O_2_ pose significant safety risks. Consequently, there is widespread interest in developing clean, cost‐effective, and renewable technologies for H_2_O_2_ production. Photocatalysts based on abundant earth elements have emerged as promising candidates for efficient H_2_O_2_ production.^[^ ^7^, ^8^, ^9^, ^10^ ^]^ Polymeric carbon nitride (CN) exhibits superior activity for H_2_O_2_ photosynthesis compared with other photocatalysts.^[^ ^11^, ^12^, ^13^, ^14^, ^15^, ^16^ ^]^ However, rapid electron–hole recombination driven by strong Coulomb forces and dielectric screening in single‐component CN photocatalysts limits photoconversion efficiency. Addressing this issue is crucial for improving H_2_O_2_ yield and fully realizing the potential of CN‐based photocatalysis.

Efforts to suppress rapid charge recombination in bulk CN often involve forming heterojunctions with secondary semiconductors or metal nanoparticles. However, these CN‐based heterojunctions often suffer from drawbacks such as metal leaching, loss of photoredox potentials, hindered interfacial charge transfer, and lattice mismatches between g‐C_3_N_4_ and the co‐catalysts.^[^ ^17^, ^18^, ^19^, ^20^, ^21^, ^22^ ^]^ To address these limitations, an emerging approach involves creating homojunctions within a single photocatalytic material to enhance interfacial photocarrier separation and improve photocatalytic efficiency.^[^ ^23^, ^24^, ^25^ ^]^ These homojunctions create internal electric fields that drive photocarrier separation, overcoming the limitations of traditional semiconductor systems.^[^ ^26^ ^]^ In contrast to conventional type‐II heterojunctions, S‐scheme electron transfer maintains a higher redox capacity in two‐component semiconductors and facilitates the efficient charge separation through the interfacial electric field.^[^ ^27^, ^28^, ^29^, ^30^, ^31^, ^32^, ^33^, ^34^, ^35^, ^36^, ^37^, ^38^, ^39^ ^]^ This approach has gained significant attention for designing and optimizing efficient charge dynamics and enhancing photocatalytic performance.^[^ ^40^, ^41^, ^42^, ^43^, ^44^, ^45^, ^46^ ^]^

**Figure 1:**
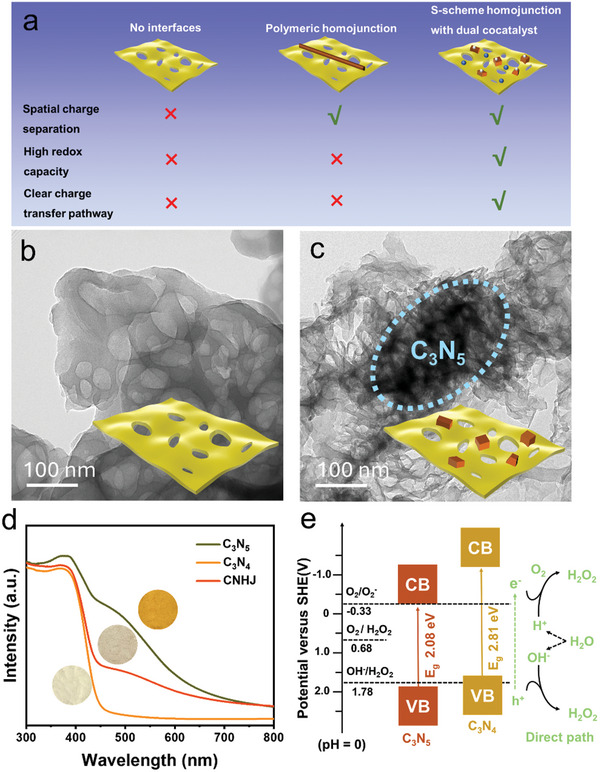
a) Schematic illustration of proposed layout. The red and green marks represent unsatisfactory and satisfactory separation efficiency, respectively. TEM images of b) C_3_N_4_ and c) CNHJ. d) UV–vis DRS spectra of C_3_N_5_, C_3_N_4_, and CNHJ. e) Energy band diagrams and H_2_O_2_ photosynthesis pathways for C_3_N_5_ and C_3_N_4_.

Developing advanced methodologies to accurately monitor the S‐scheme transfer process is essential for investigating charge transfer dynamics and optimizing charge carrier separation within CN homojunctions (**Figure** **1**a). According to the consecution of universal spatial charge separation, the dual cocatalysts loading can confirm the ultrafast and universal spatial separation and transfer of charge carriers in S‐scheme homojunction. In this study, we design a C_3_N_5_/C_3_N_4_ homojunction (CNHJ) using a two‐step calcination strategy. The resulting CNHJ photocatalyst achieves an enhanced H_2_O_2_ production rate of 8.78 mmol g^−1^ h^−1^, which is 19.1 and 2.6 times higher than that of C_3_N_5_ and C_3_N_4_, respectively. In‐depth insights into S‐scheme charge‐transfer mechanisms were obtained through state‐of‐the‐art in situ photoemission and femtosecond transient absorption spectroscopy (fs‐TAS). Density‐functional theory (DFT) calculations and in situ Fourier‐transform infrared (FTIR) spectroscopy further demonstrate that the CNHJ effectively facilitates O_2_ adsorption and activation for H_2_O_2_ photosynthesis. Consequently, by leveraging the unique advantages of S‐scheme electron transfer, the CNHJ significantly enhanced charge‐separation efficiency and improved photocatalytic H_2_O_2_ production performance.

## 2 Results and Discussion

We synthesized micro‐sized bulk C_3_N_5_ through the thermal polymerization of 3‐amino‐1,2,4‐triazole,^[^ ^47^ ^]^ using mixed precursors with varying urea content as templates for synthesizing CN homojunctions (Figure S1, Supporting Information). The micro‐morphologies of C_3_N_5_, C_3_N_4,_ and CNHJ were examined through field‐emission scanning electron microscopy and transmission electron microscopy (TEM). As shown in Figures 1b and S2 (Supporting Information), the TEM images revealed that pure C_3_N_5_ exhibited irregular and overlapping blocks.^[^ ^48^ ^]^ Pristine C_3_N_4_, synthesized through a simple thermal polymerization process using urea, displayed its characteristic layered morphology (Figure 1c). Additionally, the TEM image of CNHJ (Figure 1d) confirmed the successful formation of nanoscale C_3_N_5_ particles on the surface of porous C_3_N_4_ nanosheets, a finding further supported by N_2_‐BET analyses (Figure S3, Supporting Information).

We employed XRD and FT‐IR to examine the structural properties of C_3_N_5_, C_3_N_4_, and the homojunction (Figures S4,S5, Supporting Information). The XRD patterns highlighted the formation of graphitic carbon layers, with distinct diffraction peaks at 13.3° and 27.7°, corresponding to the (100) in‐plane structural ordering and the (002) interlayer stacking, characteristic of aromatic systems in graphitic materials. The absence of additional diffraction peaks confirmed the purity of the CN platforms. FT‐IR spectra displayed characteristic peaks ≈3100 cm^−1^ corresponded to the residual surface ─NH_2_ and/or ─OH groups, while peaks between 1200 and 1650 cm^−1^ were attributed to triazine ring stretching, and the peak at ≈810 cm^−1^ was assigned to N─H bending vibrations. UV–vis absorption spectroscopy was employed to analyze the electronic band structure of the prepared samples (Figure 1d). The introduction of C_3_N_5_ caused a color change from pale yellow to reddish brown. Compared with C_3_N_4_, these samples exhibited a redshift in the visible light absorption edge, indicating an enhanced light absorption capacity. The valence band (VB) potentials of C_3_N_5_ and C_3_N_4_ were determined to be 1.80 and 1.62 eV, respectively, as assessed through Tauc plots and UPS spectra (Figures S6,S7, Supporting Information). All conduction band (CB) levels of the polymers were above the reduction potential of O_2_, suggesting that their band structures are thermodynamically favorable for H_2_O_2_ production (Figure 1e).

**Figure 2:**
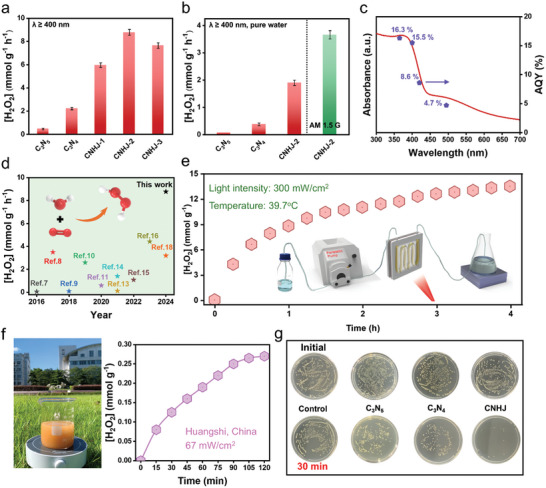
Photocatalytic H_2_O_2_ production performance for different samples in a) ethanol–water solution (10% vol.) and b) pure water, respectively. c) AQY of H_2_O_2_ production as a function of wavelength on CNHJ (purple dot), and the UV–vis absorbance spectrum (red curve). d) Activity comparison of CNHJ with other reported photocatalysts. e) Dynamic photoproduction of H_2_O_2_ over CNHJ, with an inset showing the schematic of the continuous flow photocatalytic system. the flow rate of water, 1.2 mL min^−1^; catalyst mass, 20 mg. f) Diagram of outdoor experiment and the corresponding accumulated amount of H_2_O_2_. g) Photocatalytic antibacterial properties of samples.

Under visible light irradiation (λ ≥ 400 nm), the photocatalytic H_2_O_2_ generation performance of the samples was evaluated in O_2_‐saturated ethanol solution and pure water. First, a series of control experiments were conducted to optimize reaction parameters, including catalyst dosage (Figure S8, Supporting Information), light intensity (Figure S9, Supporting Information), and reaction temperature (Figure S10, Supporting Information). As shown in **Figures** **2**a and S11 (Supporting Information), the H_2_O_2_ generation rate of C_3_N_4_ (2.22 mmol g^−1^ h^−1^) was modestly higher than that of C_3_N_5_ (0.46 mmol g^−1^ h^−1^). This enhancement is attributable to the porous and ultrathin structure of C_3_N_4_, which increased its surface interaction with O_2_. Notably, the optimal homojunction CNHJ‐2 exhibited a significantly enhanced H_2_O_2_ generation rate of up to 8.78 mmol g^−1^ h^−1^, which were 19.1 and 2.6 times higher than that of C_3_N_5_ and C_3_N_4_, respectively. At a higher ratio of urea, the moderate H_2_O_2_ generation rate (7.65 mmol g^−1^ h^−1^) might be ascribed to charge recombination at the excessive sites on the surface of CNHJ‐3 sample. Additionally, CNHJ‐2 also demonstrated a significantly improved H_2_O_2_ generation rate of up to 1.9 mmol g^−1^ h^−1^ in pure water (Figures 2b and S12, Supporting Information), which represents a 27.2‐fold and 5.1‐fold increase over C_3_N_5_ and C_3_N_4_, respectively. Furthermore, the H_2_O_2_ generation activity of CNHJ was enhanced to 3.66 mmol g^−1^ h^−1^ under AM 1.5 G irradiation in pure water (Figure 2b). Besides, a series of condition experiments about scavengers and atmosphere were conducted (Figure S13, Supporting Information). Notably, CNHJ also exhibited a lower H_2_O_2_ decomposition rate compared to both C_3_N_5_ and C_3_N_4_ under the same conditions (Figure S14, Supporting Information), indicating its superior stability during H_2_O_2_ photosynthesis. To gain further insights into the reaction kinetics, we evaluated the first‐order kinetics formation rate (K*_f_*) and the zero‐order kinetics decomposition rate (K*_d_*), through the analysis of H_2_O_2_ formation and decomposition data. As shown in Figure S15 (Supporting Information), CNHJ demonstrated the highest K*_f_* and a relatively lower K*_d_*, which together contributed to its superior performance in H_2_O_2_ production. Wavelength‐dependent photocatalytic tests (Figure 2c) revealed the apparent quantum yield (AQY) of 15.5% at 400 nm for CNHJ, as determined using a custom‐built light‐emitting diode (LED) light source. As illustrated in Figure 2d, compared with currently developed CN‐based photocatalysts, CNHJ exhibited superior performance in terms of H_2_O_2_ yields, which approached the high values reported for powder photocatalysts. The cyclic experiments were conducted and the results are exhibited in Figure S16 (Supporting Information). Obviously, the yield of H_2_O_2_ can still reach to 8.52 mmol g^−1^ h^−1^ after six consecutive cycles.

To assess the long‐term catalytic stability and scalability of photocatalytic H_2_O_2_ production, the photocatalyst was employed in the serpentine channel of a custom‐designed flow‐type quartz microreactor equipped with a peristaltic pump (Figure 2e inset). After a 4‐h reaction period, the accumulated H_2_O_2_ concentration reached 13.5 mmol g^−1^, significantly exceeding the practical application threshold for H_2_O_2_ applications. Additionally, X‐ray diffraction (XRD) analysis of the CNHJ after photocatalytic reaction revealed no significant structural changes (Figure S17, Supporting Information). These results indicate that the catalyst demonstrated exceptional stability and holds considerable potential for practical applications. To evaluate the conversion of solar energy to chemical energy under realistic conditions, the H_2_O_2_ production performance of CNHJ was assessed under natural light (Figure 2f). Upon exposure to natural sunlight for 120 min, the accumulated H_2_O_2_ concentration reached ≈0.27 mmol g^−1^, showcasing the potential of CNHJ for solar‐driven H_2_O_2_ production in real‐world environments. In situ photocatalytic degradation experiments were conducted to further assess the practical applicability of the designed catalyst (Figure S18, Supporting Information). The CNHJ system demonstrated effective degradation of organic pollutants. Additionally, disinfection experiments were conducted on *Escherichia coli* using photocatalysis to explore potential biomedical applications (Figure 2g).^[^ ^1^ ^]^ Compared with the control experiment, the CNHJ system demonstrated excellent bactericidal efficacy, achieving nearly complete elimination of the bacteria within 30 min. This rapid disinfection can be attributed to the synergistic effects of photocatalytic oxidation and the concurrent generation of H_2_O_2_.

**Figure 3:**
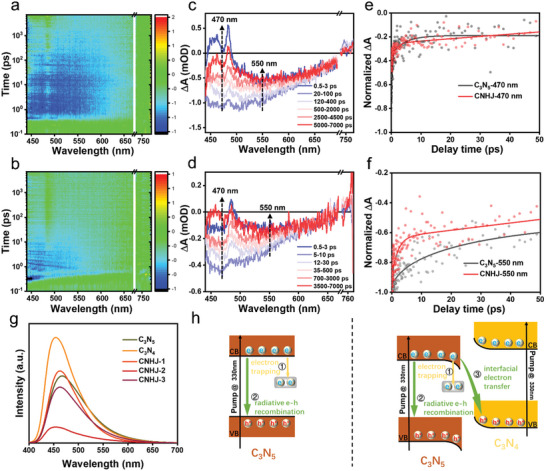
2D pseudocolor transient absorption (TA) spectra of a) C_3_N_5_ and b) CNHJ using a 340‐nm pump pulse. TA spectra of c) C_3_N_5_ and d) CNHJ at the indicated time delays. TA kinetics measured at e) 470 nm and f) 550 nm. g) Photoluminescence (PL) spectra of C_3_N_5_, C_3_N_4_, and CNHJ composites. h) Schematics of the electron quenching pathways in the C_3_N_5_ and CNHJ homojunctions.

To gain a comprehensive understanding of electron‐transfer dynamics within the CNHJ, femtosecond transient absorption spectroscopy (fs‐TAS) was meticulously performed. The observed negative peak at 470 nm, near the absorption edge of C_3_N_5_, indicated the ground‐state bleach (GSB) of electrons in the CB of C_3_N_5_ (**Figure** **3**a–f). Additionally, the broad negative peak spanning 500–750 nm was related to the dynamics of electrons in the shallow trap states of pristine C_3_N_5_ (Figure 3c,d). The presence of these electron trap states was confirmed by the photoluminescence (PL) spectra of C_3_N_5_, C_3_N_4_, and CNHJ composites (Figure 3g). The pronounced PL peak ≈470 nm corresponded to the band‐to‐band recombination of electron–hole pairs. Moreover, the composite exhibited lower PL intensity and reduced GSB peak intensity than pristine C_3_N_5_, indicating that the incorporation of C_3_N_4_ introduced a new electron transfer pathway, thereby suppressing charge recombination. The transient absorption (TA) kinetics of C_3_N_5_ and the CNHJ composite, probed at 470 nm, were fitted using double‐exponential and triple‐exponential functions, respectively (Figure 3e). For single C_3_N_5_, the shorter lifetime τ_1_ (0.29 ps) reflected the rapid electron trapping process, corresponding to the relaxation of photogenerated electrons from the CB to shallow trap states within C_3_N_5_.^[^ ^49^ ^]^ The longer lifetime τ_2_ (4.55 ps) was due to the band‐to‐band recombination of photogenerated electrons and holes within C_3_N_5_. For the TA kinetics of the CNHJ composite probed at 470 nm, the values of τ1 (0.44 ps) and τ_2_ (3.79 ps) were similar to those observed for C_3_N_5_, suggesting comparable electron transfer kinetics. The additional τ_3_ (1.56 ps) was likely due to interfacial electron transfer from the CB of C_3_N_5_ to the VB of C_3_N_4_, highlighting the enhanced charge separation within the CNHJ. Additionally, the dynamics of electrons in the trapping states probed at 460 nm are shown in Figure 3f. The ratio of bleaching signal intensities (R_b_) at 470 and 550 nm serves as a concise indicator of electron populations in the CB and the shallow defect states.^[^ ^49^ ^]^ The CNHJ composite, with an R_b_ value of 2.09 at 550 nm, exhibited a higher R_b_ than the 1.97 recorded for pristine C_3_N_5_, indicating a reduction in electron trap states within the composite. Additionally, the shorter τ_2_ and the average electron lifetime (τ_a_) for the CNHJ indicate that the S‐scheme heterojunction contributed to reduced photogenerated carrier recombination compared with pristine C_3_N_5_ (Figure 3h). This observation is further supported by the TA kinetics at 550 nm. The charge carrier generation and mobility of CNHJ were further investigated through photocurrent measurements and electrochemical impedance studies.^[^ ^50^ ^]^ The photocurrent measurements revealed that CNHJ exhibited the highest photocurrent density among all the samples (Figure S19, Supporting Information), reflecting improved the photogenerated charge separation and transfer efficiency during photoexcitation. Additionally, CNHJ showed a smaller arc radius and lower charge‐transfer resistance than pristine C_3_N_5_ and C_3_N_4_, further corroborating the enhanced charge mobility in the homojunction (Figure S20, Supporting Information).

**Figure 4:**
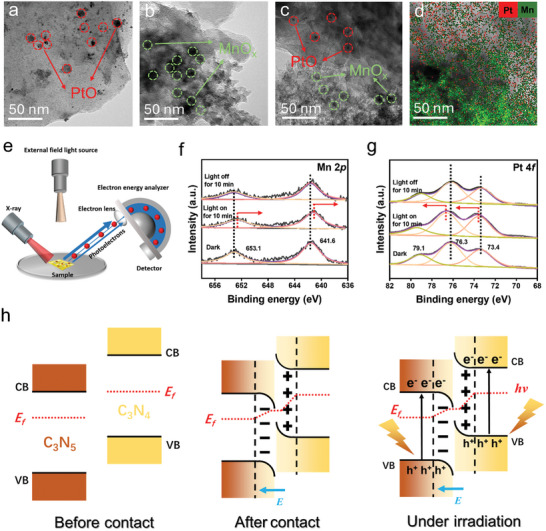
TEM images: a) C_3_N_5_@Pt, b) C_3_N_4_@Mn, and c) CNHJ@PtMn. d) HAADF–STEM image with coupled elemental mapping of CNHJ@PtMn. e) Schematic of the in situ illuminated XPS measurement. XPS spectra: f) Mn 2*p* and g) Pt 4*f* of CNHJ@PtMn under various conditions. h) Formation of the CNHJ S‐scheme heterojunction and the proposed charge transfer and separation mechanism.

To further elucidate the direction of electron transfer, both ex situ and in situ irradiated X‐ray photoelectron spectroscopy (XPS) analyses were performed. However, verifying the charge transfer pathways in homojunctions through in situ XPS can be challenging due to the elemental similarity of the components in the homojunction. To address this, metallic cocatalysts, which provide a substantial number of free electrons, can serve as effective indicators for detecting the loss and gain of electron transfer in homojunctions (Figure S21, Supporting Information). In this study, bimetallic cocatalysts, including PtO_x_ and MnO_x_, were deposited onto the homojunction to monitor the electron transfer process through in situ XPS under external field illumination (**Figure** **4**e).^[^ ^51^ ^]^ When X‐rays irradiate the sample surface, the excited electrons will escape from the sample surface and enter the analyzer. The morphology of C_3_N_5_@Pt, C_3_N_4_@Mn, and CNHJ@PtMn is depicted in Figure 4a–c. PtO nanoparticles and MnO_x_ flakes occurred on the surfaces of C_3_N_5_ and C_3_N_4_, respectively (Figures S22,S23, Supporting Information). According to the energy‐dispersive spectrometry spectra shown in Figure 4d, PtO_x_ was predominantly concentrated on C_3_N_5_, while MnO_x_ was mainly concentrated on C_3_N_4_.

The binding energies of Mn 2*p*1/2 (653.1 eV), Mn 2*p*3/2 (641.6 eV), Pt 4*f*5/2 (79.1 eV), Pt 4*f*5/2 (76.3 eV), and Pt 4*f*7/2 (73.4 eV) indicated that Mn existed in a valence state between +3 and +4, while Pt predominantly exhibited a +2 oxidation state, similar to that in PtO_x_. Compared with its original state, the binding energy of Mn 2*p* blue‐shifted by 0.5 eV after 10 min of illumination and then reverted to the “dark‐state” binding energy once the light was turned off (Figure 4f). This decrease in the binding energy of Mn 2*p* under illumination suggests that MnO_x_ gains electrons. Specifically, photogenerated electrons in the CB of C_3_N_5_ were transferred to the Mn 2*p* orbital in C_3_N_4_, driven by the electric field generated at the interface. This observation supports the role of MnO_x_ as an effective electron acceptor. Interestingly, the binding energy changes of Pt 4*f* on C_3_N_5_ showed an inverse relationship with those of Mn 2*p*. The binding energy of Pt 4*f* red‐shifted by 0.7 eV under light illumination compared with its value in the dark. The binding energy of Mn 2*p* and Pt 4*f* fully returned to the “dark‐state” energy position after the light was turned off (Figure 4g).^[^ ^51^ ^]^ This persistent shift suggests that photogenerated holes remain trapped in PtO_x_, which acts as an oxidizing cocatalyst. As illustrated in the schematic (Figure S21, Supporting Information), the process involved indirect electron transfer from PtO_x_ to MnO_x_ anchored on C_3_N_4_, driven by the interfacial electric field.

To further substantiate that photogenerated electrons at the interface of a polymeric homojunction adhere to the S‐scheme transfer mechanism, light‐assisted Kelvin probe force microscopy (KPFM) was utilized to investigate alterations in the local potential of the homojunction before and after illumination (Figure S24, Supporting Information). As illustrated in Figure S24 (Supporting Information), the distribution of the two‐phase surface potential in the absence of light reveals that the surface potential of C_3_N_5_ in the homojunction exceeds that of C_3_N_4_, resulting in a potential difference of 6.2 mV. Moreover, the work function of C_3_N_5_ is greater than that of C_3_N_4_. Upon exposure to light, there is a notable alteration in the surface potential at the junction of C_3_N_5_ and C_3_N_4_ within the same region. The observed changes in the KPFM surface potential of the C_3_N_5_/C_3_N_4_ homojunction, induced by light irradiation, provide a direct visualization of the dynamic transfer of photogenerated electrons from C_3_N_5_ to C_3_N_4_, captured in both real‐space and real‐time dimensions. This charge transfer pathway suggests the formation of an S‐scheme homojunction between C_3_N_5_ and C_3_N_4_ (Figure 4h), enhancing the photocatalytic activity of the CNHJ system, further validating its potential for efficient solar energy conversion and practical applications in H_2_O_2_ production.

**Figure 5:**
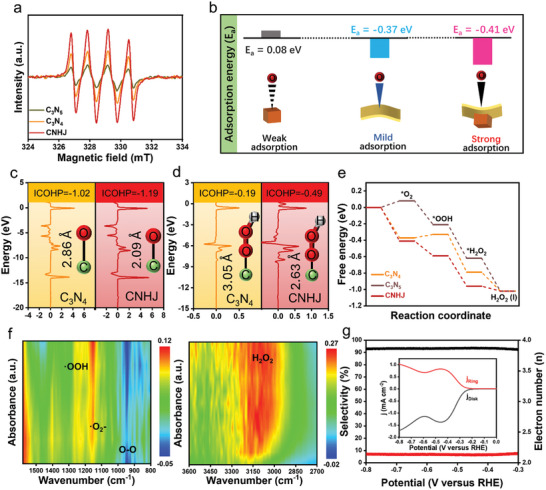
a) EPR spectra and b) adsorption energies of C_3_N_5_, C_3_N_4_ and CNHJ. COHP analyses of c) O_2_ adsorption and d) ^*^OOH adsorption on C_3_N_4_ and CNHJ. (C: green, O: red, H: gray). e) Free energy diagram for O_2_ conversion to H_2_O_2_ over C_3_N_4_, C_3_N_5_ and CNHJ. f) 2D pseudocolor in situ DRIFT spectra of CNHJ. g) Electron transfer numbers and H_2_O_2_ selectivity at different potentials were calculated from RRDE data of CNHJ (inset of RRDE curves).

To elucidate the relationship between the structural evolution of S‐scheme homojunctions and high H_2_O_2_ yield, both experimental and theoretical approaches were employed to probe the surface reaction mechanisms. Quenching experiments and electron paramagnetic resonance (EPR) measurements were conducted to identify potential active species within the photocatalytic system. As shown in **Figures** **5**a and S25 (Supporting Information), continuous N_2_ bubbling significantly suppressed H_2_O_2_ production, indicating that H_2_O_2_ generation mainly resulted from the photocatalytic reduction of O_2_. Furthermore, the addition of benzoquinone (BQ), a superoxide quencher, completely inhibited the photocatalytic H_2_O_2_ production in the CNHJ system, confirming that superoxide radicals (·O_2_^−^) played a crucial role in H_2_O_2_ generation. As shown in Figures 5a and S26 (Supporting Information), significant ·O_2_^−^ signals were detected under illumination with 5,5‐dimethyl‐1‐pyrroline N‐oxide as a spin trap for EPR measurements.^[^ ^52^ ^]^ These findings suggest that the initial step of the oxygen reduction reaction (ORR) involved the adsorption and activation of O_2_.^[^ ^53^ ^]^ The adsorption configuration of O_2_ on the catalyst can be classified into three fundamental types (Figure S27, Supporting Information): Yeager type, Griffith type, and Pauling type. According to the identification of different adsorption sites shown in Figure S28 (Supporting Information), O_2_ adsorbed at the C site on the C_3_N_4_ layer in CNHJ exhibited an end‐on configuration (O_1_─C─N_3_), differing from the adsorption behavior observed in pristine C_3_N_5_ and C_3_N_4_ (Figures 5b and S29, Supporting Information).^[^ ^54^, ^55^ ^]^ Additionally, the O_2_‐TPD analysis revealed that the CNHJ sample exhibited the highest pronounced O_2_ desorption response among all the samples investigated, indicating its superior O_2_ adsorption capacity (Figure S30, Supporting Information).

This enhanced O_2_ adsorption capability was further supported by crystal orbital Hamilton population (COHP) calculations, which showed a lower integrated COHP value (−1.40) for C─O_ads_ bonds in CNHJ compared with C─O_ads_ bonds in pristine C_3_N_4_ (−1.38), as well as a shorter bond length (2.09 Å) than in pristine C_3_N_4_ (2.86 Å). These results indicate stronger C─O_ads_ bonding at the surface C sites of CNHJ (Figure 5c).^[^ ^56^ ^]^ To investigate the impact of enhanced O_ads_ bonds on the formation of the OOH_ads_ intermediate in the CNHJ catalyst, COHP calculations were performed. As shown in Figure 5d, CNHJ exhibited both a lower COHP value (−1.66) and a shorter bond length (2.64 Å, inset) of C─OOH_ads_ compared with pristine C_3_N_4_ (−0.19 and 3.05 Å, respectively). These results demonstrate that CNHJ exhibited stronger OOH_ads_ bonds, which is beneficial for improving the selectivity of photocatalytic H_2_O_2_ production. The O_2_ adsorption energy on CNHJ was also reduced from −0.37 eV in pristine C_3_N_4_ to −0.41 eV, suggesting that O_2_ adsorbs more easily on CNHJ (Figure 5b–e). As shown in Figures 5e and S31, and Table S1 (Supporting Information), the formation of ^*^OOH was the rate‐determining step in the overall ORR (^*^+O_2_→^*^O_2_→^*^OOH→^*^HOOH→H_2_O_2_+^*^), as it involved overcoming an energy barrier on pristine C_3_N_4_.^[^ ^57^, ^58^ ^]^ CNHJ had a lower energy barrier of −0.46 eV for ^*^OOH formation in the ORR, compared with 0.04 eV for pristine C_3_N_4_, facilitating more efficient H_2_O_2_ generation.

In situ, DRIFT spectroscopy measurements were conducted to elucidate the mechanism of photocatalytic H_2_O_2_ generation over CNHJ. The DRIFT analysis of CNHJ was performed in a confined space after a 60‐min purge with steam‐saturated O_2_ flow. The spectra exhibited peaks at 1251, 1149, and 943 cm^−1^ (Figures 5f and S31, Supporting Information), corresponding to the OOH intermediate species,─O_2_^−^, and O─O stretching vibration, respectively.^[^ ^59^ ^]^ Additionally, a broad peak at 3109 cm^−1^, indicative of H_2_O_2_ formation, was observed. These results confirm that·O_2_^−^ species, formed via the adsorption of single‐molecule O_2_, play a key role in H_2_O_2_ generation. Furthermore, the ORR activity and selectivity of CNHJ for electrocatalytic H_2_O_2_ production were investigated in an O_2_‐saturated 0.10 M phosphate buffer solution (PBS, pH 7) through rotating ring‐disk electrode (RRDE) measurements. The ORR current (depicted by the black line) and the H_2_O_2_ oxidation current (depicted by the red line) were measured on the disk electrode and the platinum ring electrode, respectively, as shown in the inset of Figure 5g. Additionally, the number of transferred electrons (*n*) during the reaction process determined the selectivity for H_2_O_2_. For CNHJ, this value was calculated as 2.13, indicating a high H_2_O_2_ selectivity of ≈93.2% via a two‐electron ORR (Figure 5g).^[^ ^60^ ^]^ RRDE measurements further demonstrated that CNHJ exhibited low overpotential and high current density, highlighting the significant potential for electrocatalytic H_2_O_2_ synthesis in neutral electrolytes.

## 3 Conclusion

In summary, we synthesized a CN homojunction as CNHJ to elucidate the interfacial electron‐transfer mechanism relevant to artificial photosynthesis for H_2_O_2_ production. The CNHJ exhibited improved O_2_ adsorption properties, which facilitated rapid H_2_O_2_ production kinetics. Consequently, an exceptional H_2_O_2_ production rate of 8.78 mmol g^−1^ h^−1^ was achieved, which was 19.1 and 2.6 times those achieved by C_3_N_5_ and C_3_N_4_, respectively. Additionally, CNHJ exhibited high catalytic performance in a flow photocatalytic device, showcasing its potential for effective photocatalytic disinfection applications. A combination of theoretical simulations and experimental characterization demonstrated that photogenerated electrons at the homojunction interface underwent S‐scheme transfer from C_3_N_5_ to C_3_N_4_. Specifically, the S‐scheme electron transfer was monitored in situ using XPS, employing a bimetallic cocatalyst as a probe to further confirm the transfer dynamics. Overall, this study not only elucidates the dynamic carrier transfer mechanism of S‐scheme homojunctions but also proposes a novel design strategy for materials intended to enhance the efficiency of H_2_O_2_ photosynthesis. The insights gained here may pave the way for future advancements in photocatalytic technologies and their applications in sustainable energy solutions.

## Conflict of Interest

The authors declare no conflict of interest.

## Supporting information



Supporting Information

## Data Availability

The data that support the findings of this study are available from the corresponding author upon reasonable request.
