# Supplementary material for: Verifying the Unique Charge Migration Pathway in Polymeric Homojunctions for Artificial Photosynthesis of Hydrogen Peroxide
Source: Adv Sci (Weinh). 2025 Mar 5;12(16):2500218. doi: 10.1002/advs.202500218 (PMC12021120; doi:10.1002/advs.202500218)
Supplement: Supplementary file 1 — Supporting Information [file ADVS-12-2500218-s001.docx]

Supporting Information

Verifying the Unique Charge Migration Pathway in Polymeric Homojunctions for Artificial Photosynthesis of Hydrogen Peroxide

Qiang Cheng, Jingping Li, Yuxin Huang, Xiufan Liu, Biao Zhou, Qiao Xiong and Kai Wang*

***Experimental Section***

*Materials*: 3-amino-1,2,4-triazole (AR), urea ((NH_2_)_2_CO, AR), chloroplatinic acid hexahydrate (H_2_PtCl_6_·6H_2_O, AR), potassium permanganate (KMnO_4_, AR), sodium borohydride (NaBH_4_, AR), ethanol (EtOH, AR), tert-butanol (TBA, AR), p-benzoquinone (pBQ, AR) and tetracycline (TC, AR) were all purchased from Sinopharm Chemical Reagent Co., China, Sigma-Aldrich Chemical Reagent Co., China or Alfa Aesar. Ultrapure water (18.20 MΩ) was used in all experiments.

*Synthesis of C_3_N_4_*: Urea (10 g) was placed in a ceramic crucible and calcined at 520 °C under Ar atmospheres for 3 h with a heating rate of 5 °C min^−1^. The obtained product was ground, washed, and centrifuged, followed by drying at 60 °C for 8 h.

*Synthesis of C_3_N_5_*: 1.5 g of 3-amino-1,2,4-triazole was added to a ceramic crucible and heated to 550 °C under Ar atmospheres for 6 h at a heating rate of 2.5 °C min^−1^. The obtained product was ground, washed, and centrifuged, followed by drying at 60 °C for 8 h.

*Synthesis of carbon nitride homojunction (CNHJ)*: C_3_N_5_ (0.15 g) was added into 4 mL of deionized water to be ultrasonically stirred for 1 h to completely disperse the C_3_N_5_ and a specific quantity of urea (with weight ratios of urea to C_3_N_5_ as 5:1, 10:1, and 20:1) were added. The resulting mixture was then transferred to water bath and stirred while evaporating the solvent at 85 °C. Liquid nitrogen was added into the mixture to freeze it to ice during stirring. Subsequently, the ice was lyophilized in a vacuum freeze dryer. The above precursors were heated to 520 °C under Ar atmospheres for 3 h at a heating rate of 2.5 °C min^−1^. The obtained product was ground, washed, and centrifuged, followed by vacuum drying at 60 °C for 8 h.

*Synthesis of C_3_N_5_-supported PtO_x_ (C_3_N_5_@Pt)*: 100 mg of C_3_N_5_ was dispersed in 200 mL methanol. Then, 13.3 mL of chloroplatinic acid solution (1 mg/mL) was added, and the Pt species were deposited on C_3_N_5_ by photoreduction. The obtained product was ground, washed, and centrifuged, followed by vacuum drying at 60 °C for 8 h. The resulting product was marked as C_3_N_5_@Pt.

*Synthesis of C_3_N_4_-supported MnO_x_ (C_3_N_4_@Mn)*: 30 mL of distilled water and 30 mL of ethanol were mixed and 100 mg of C_3_N_4_ was then added into above mixed solution and stirred for 30 min. 6.96 mg of KMnO_4_ was also added into a beaker and stirred using a magnetic stirrer until it was completely dissolved. Next, 37.8 mg of NaBH_4_ was added into the ethanol solution. Finally, the KMnO_4_ solution was added dropwise into above mixed solution and stirred for another 24 h. The resulting product was marked as C_3_N_4_@MnO_x_

*Synthesis of carbon nitride homojunction supported with bimetallic cocatalysts (CNHJ@PtMn)*: C_3_N_5_@Pt and C_3_N_4_@Mn were mixed and stirred according to the mass ratio of 1:4. The obtained product was ground, washed, and centrifuged, followed by vacuum drying at 60 °C for 8 h. The resulting product was marked as CNHJ@PtMn.

***Characterizations***

The structure and crystallinity of composites were analyzed by powder X-ray diffraction (XRD) on a D/MAX-RB diffractometer with Cu Kα radiation. Transmission electron microscopy (TEM) images were collected on a JEM-2100 transmission electron microscope (JEOL) with an acceleration voltage of 200 kV. Fourier-transform infrared (FTIR) spectra were recorded on a Nicolet iS5 spectrometer (Thermo) using the KBr pellet technique. X-ray photoelectron spectroscopy (XPS) was performed on a Thermo Scientific ESCALA 210 XPS spectrometer system (USA) with 300 W Al Kα radiation to survey the elemental composition and valence states of these photocatalysts. Ultraviolet photoelectron spectroscopy (UPS) was measured with an ESCALAB Xi+ X-ray photoelectron energy dispersive spectrometer (Thermo). EPR measurements were obtained using a Bruker model EMXPLUS 10/12 spectrometer. In-situ diffuse reflectance infrared Fourier transform spectrometer (INVENIO S, Bruker) equipped with a liquid N_2_ cooled MCT detector and a high-temperature reaction chamber (Praying Mantis, Harrick). Femtosecond transient absorption spectra of the as-prepared photocatalysts were obtained on a pump-probe system (Helios, Ultrafast System) with a maximum time delay of ~8 ns using a motorized optical delay line under ambient conditions. The 330 nm-pump pulses (600 μW average at tested samples) were generated by the 1 kHz regenerative amplifier (Coherent Libra, 800 nm, 35 fs, 5 mJ) in an optical parametric amplifier (OPerA Solo), seeded with a mode-locked Ti: sapphire oscillator (Coherent Vitara, 800 nm, 80 MHz) and pumped with a laser (Coherent Evolution-50C, 1 kHz system).

***Photocatalytic H_2_O_2_ production***

Photocatalytic H_2_O_2_-production activity was examined in an O_2_-saturated aqueous solution with ethanol as a hole scavenger. The reactor was irradiated using a 300 W xenon lamp (50-500 mW cm^-2^, PLS-SXE300+/UV, Beijing Perfectlight) while stirring at 10–30 °C. The H_2_O_2_ generated in the reaction was determined by iodometry method. During the photocatalytic test, 1 mL of reaction mixture sampled and percolated by a Millipore filter was added to 1 mL of 0.4 M potassium iodide (KI) aqueous solution and 1 mL of 0.1 M C_8_H_5_KO_4_ aqueous solution. The concentration of H_2_O_2_ was estimated by using an UV-vis spectrophotometer (UV-1240, Japan) to measure the absorbance of the wavelength at 350 nm. The AQY of photocatalytic H_2_O_2_ production was measured using different bandpass filters and calculated according to Eq. (1):

AQY$=\frac{Number of reacted electrons}{Number of incidented of photons}\times100\%=\frac{2nN_{A}hc}{SPt\lambda}\times100\%$

where n is the amount of H_2_O_2_ molecules (mol), NA is the Avogadro constant (6.022×10^23^ mol^-1^), h is the Planck constant (6.626 × 10^-34^ JS), c is the speed of light (3×10^8^ m s^-1^), S is the irradiation area (cm^2^), P is the intensity of irradiation light (W cm^-2^), t is the photoreaction time (s), λ is the wavelength of the monochromatic light (m).

***Photocatalytic decomposition of H_2_O_2_***

The decomposition of H_2_O_2_ was carried out in a 50 mL beaker. Typically, 5 mg of photocatalyst was dispersed in a 10 mL H_2_O_2_ solution (2 mM), and the decomposition experiment was conducted under dark conditions. During the reaction process, 1 mL of suspension was collected every 10 min and filtered through a 0.22 μm membrane to remove the photocatalyst. The H_2_O_2_ concentration was determined using the iodometry method.

***Photocatalytic degradation measurements***

Photocatalytic degradation of pollutants was conducted in a photochemical reactor. Typically, 20 mg of catalyst was dispersed in 100 mL pollutant solutions (RhB:10 mg L^-1^; MB:10 mg L^-1^; TC:20 mg L^-1^) by ultrasonication. The resulting solution was stirred in the dark for 10 min to ensure adsorption-desorption equilibrium. The reaction temperature was maintained at 25 °C using an external condenser, and the photocatalytic reaction was performed using a 300 W xenon lamp (PLS-SXE300D, Beijing Perfectlight) as the light source. During the reaction process, 0.5 mL of suspension was collected every 5 min and filtered through a 0.22 μm membrane to remove the catalyst. The concentrations of contaminants were detected on an UV–visible spectrophotometer.

***Photocatalytic disinfection performance***

*Escherichia* coli (*E*. coli) was selected as the model pathogenic bacterium for the antibacterial activity test. An equal volume of *E*. coli lyophilized solution was inoculated into LB broth and cultured at 37 °C in a shaking incubator (180 rpm) for 10 h to obtain the activated bacterial strains. In the photocatalytic antibacterial experiment, a sterilized catalyst (10 mg) was placed in a 20 mL quartz test tube containing a 10 mL diluted bacterial solution (A mixed bacterial solution without the catalyst served as a control). The test tubes were then sealed and placed under a light source for photocatalytic antibacterial treatment. The suspension samples were collected every 10 min, the H_2_O_2_ yield was measured, and the samples were diluted 1000-fold with a PBS solution. The diluted suspension (100 μL) was spread on agar plates and incubated at 37 °C for 18 h.

***Photoelectrochemical characterizations***

The photoelectrochemical properties were assessed using an electrochemical workstation (CHI 760E, China) within a three-electrode system. The working electrode was prepared by applying the photocatalyst onto a 1.0 cm^2^ FTO glass substrate. The Ag/AgCl (in a saturated KCl solution) and Pt foil were designated as the reference and counter electrodes, respectively. The PEC assessments were carried out in a 0.5 M Na_2_SO_4_ solution using a 300 W Xenon arc lamp for illumination. All electrochemical experiments were conducted at room temperature and without any special aeration treatment. Additionally, in a typical three-electrode cell, the oxygen reduction reaction (ORR) on various catalysts was monitored using a rotating ring-disk electrode (RRDE). In a phosphate buffer solution of pH 6.9 and 0.1 M, the Ag/AgCl electrode was the reference electrode, while the Pt plate acted as the counter electrode. The working electrode consisted of an RRDE comprising a glassy carbon disk and a platinum ring. To prepare a working electrode with a catalyst layer, 6 µL of the prepared slurry was applied onto the glassy carbon disk and then subjected to vacuum drying.

The H_2_O_2_ production selectivity was calculated according to Eq. (2):

H_2_O_2_(%)$=\frac{2\times i_{R}}{N\times\left| i_{D}\left| + \right. \right.i_{R}}\times100$

The electron transfer number (n) was calculated according to Eq. (3):

$$n=\frac{4 \left| i_{D}\left| \right. \right.}{\left| i_{D}\left| + \frac{i_{R}}{N} \right. \right.}$$

where i_R_ and i_D_ are the ring and disk currents, respectively, and N is the collection efficiency of the RRDE (N = 0.25).

***Computational methods***

The electronic and catalytic properties of pristine C_3_N_5_, C_3_N_4_, and CNHJ were investigated by using the Vienna Ab-initio Simulation Package with the revised Perdew–Burke–Ernzerhof functional of the generalized gradient approximation. The projector-augmented wave pseudopotential was used to simulate the interaction between ionic and valence electrons. A supercell containing 4 [C_3_N_4_] units was used to simulate C_3_N_4_ photocatalyst. The experimentally confirmed C_3_N_5_ structure was built from C_3_N_4_ layer. The geometry optimization was conducted with the cutoff energy of 1 × 10^-4^ eV and energy convergence of 400 eV at the gamma point. After geometry optimization, the projected density of states (PDOS) and charge density mappings were calculated with a Monkhorst–Pack k-point mesh of 2 × 2 × 1, a cutoff energy of 1 × 10^-5^ eV and an energy convergence of 400 eV were used for oxygen adsorption. The adsorption energy E_ads_ was defined as E_ads_ = E_total_-E_surface_-E_O2_, where E_total_, E_surface_, and E_O2_ represent the energy of adsorption configurations, the energy of metallic surfaces, and the energy of molecular O_2_, respectively. In addition, the ΔG of HOO∗ intermediate on the surface was calculated by the equation G = E + ZPE-TS, where E is the total energy, ZPE is the zero-point energy, T is the temperature (298.15 K), and S is the entropy. Several configurations of the adsorbed models were considered in the simulation, and the most favorable ones are presented based on the adsorption energy.


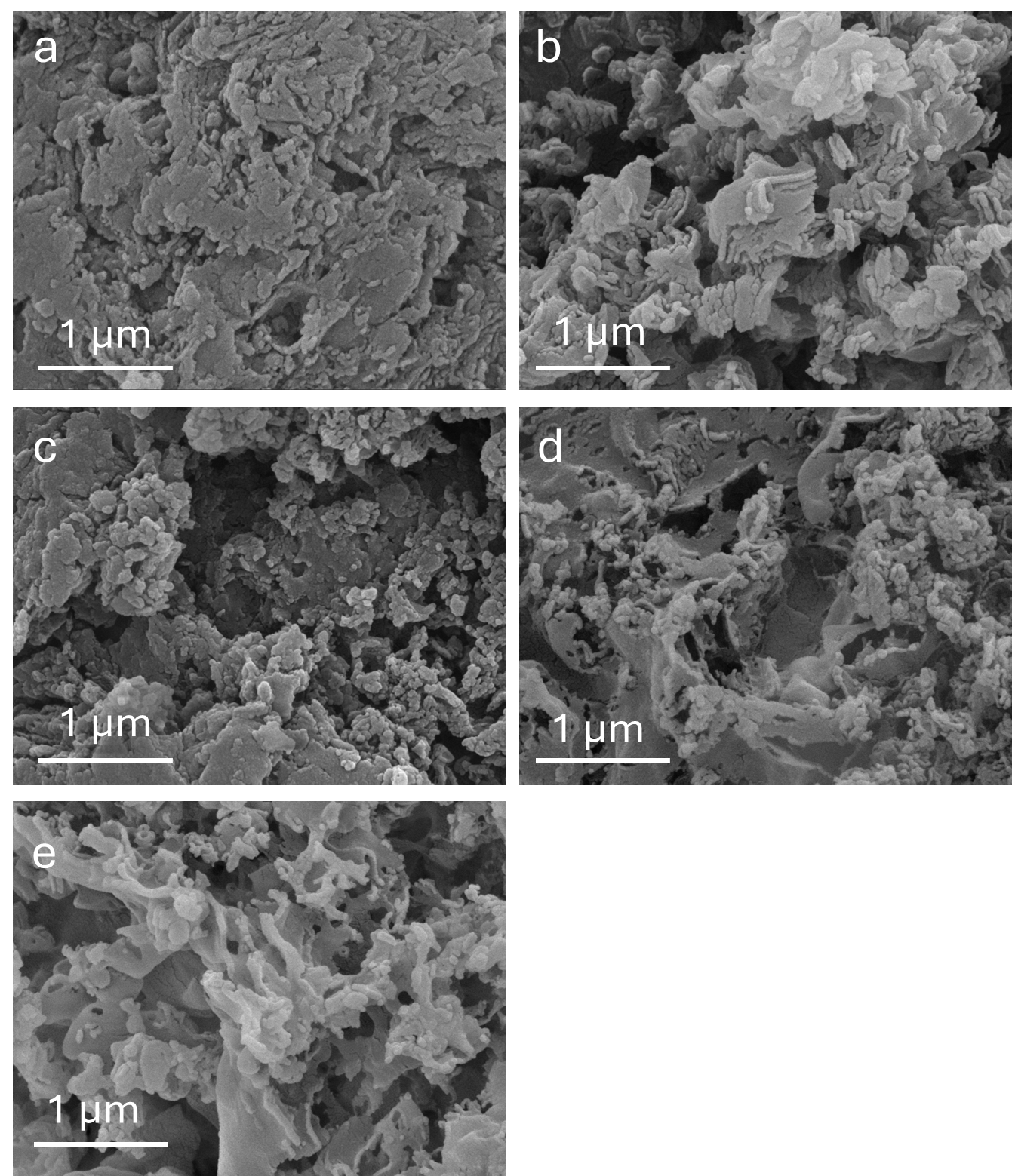


**Figure S1** FESEM images of (a) C_3_N_5_, (b) C_3_N_4_, (c) CNHJ-1, (d) CNHJ-2, (e) CNHJ-3.


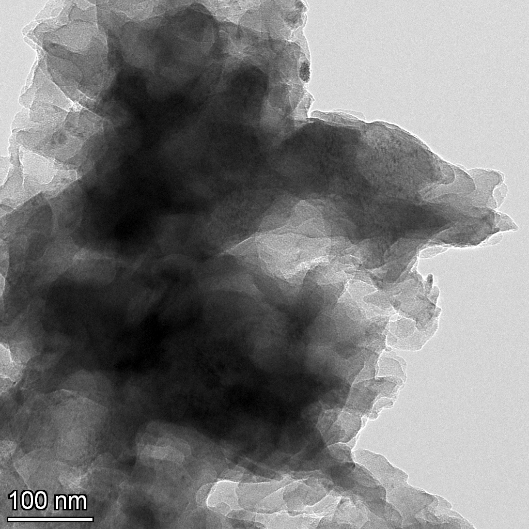


**Figure S2** TEM image of C_3_N_5_.


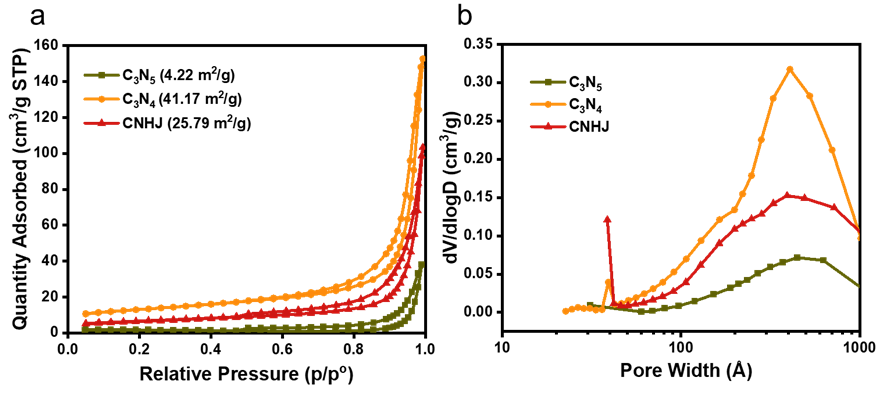


**Figure S3** (a) N_2_ sorption isotherms and (b) pore size distribution of C_3_N_5_, C_3_N_4_ and CNHJ.

**Figure S4** XRD patterns of C_3_N_5_, C_3_N_4_, CNHJ-1, CNHJ-2 and CNHJ-3.

**Figure S5** FT-IR spectra of C_3_N_5_, C_3_N_4_, CNHJ-1, CNHJ-2 and CNHJ-3.

**Figure S6** Tauc plots of C_3_N_5_ and C_3_N_4_.

*
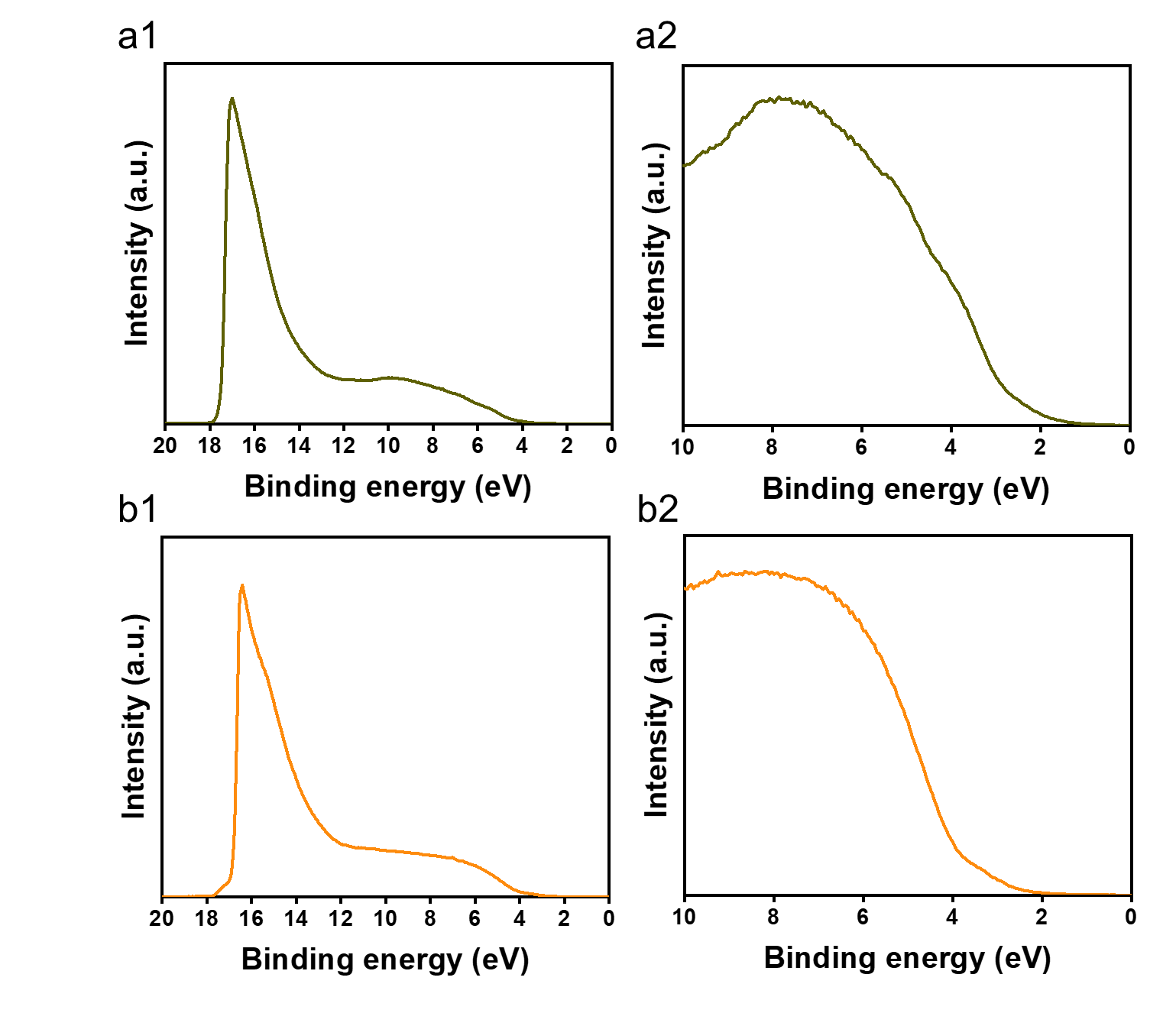
*

**Figure S7** UPS spectra of (a) C_3_N_5_ and (b) C_3_N_4_.

**

**Figure S8** The photocatalytic performance of CNHJ as a function of dosage of catalyst.

**

**Figure S9** The photocatalytic performance of CNHJ as a function of light intensity.

**

**Figure S10** The photocatalytic performance of CNHJ as a function of temperature.

**Figure S11** The time course of H_2_O_2_ production measured in ethanol-water solution (10% vol.) under visible light irradiation.

**

**Figure S12** The time course of H_2_O_2_ production measured in pure water under visible light irradiation.

**Figure S13** Photocatalytic H_2_O_2_ production over CNHJ under different conditions.

**

**Figure S14** H_2_O_2_ decomposition curves of samples under light irradiation (C_0_=2 mM H_2_O_2_, N_2_ purging).

*
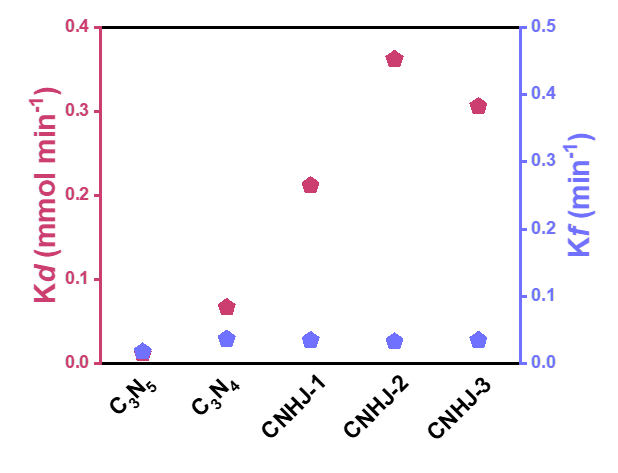
*

**Figure S15** Photocatalytic H_2_O_2_ formation and decomposition rate constant of samples.

**Figure S16** Cycling runs for the photocatalytic H_2_O_2_ production over CNHJ-2.

**Figure S17** XRD patterns of CNHJ after the photocatalytic reaction.


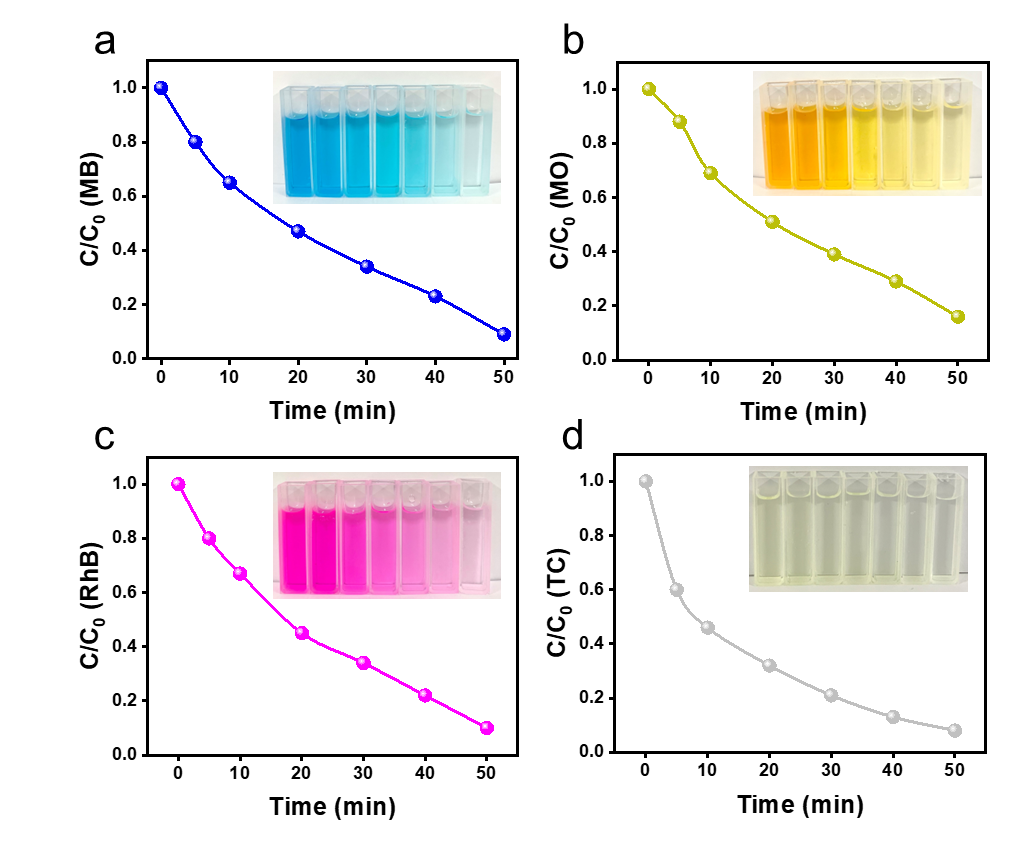


**Figure S18** Photocatalytic degradation performances of (a) Methylene blue, (b) Methyl orange, (c) Rhodamine B and (d) tetracycline over CNHJ as the photocatalysts.

**Figure S19** Photoelectrochemical responses of C_3_N_5_, C_3_N_4_, and CNHJ.

**Figure S20** EIS plots of C_3_N_5_, C_3_N_4_, and CNHJ.


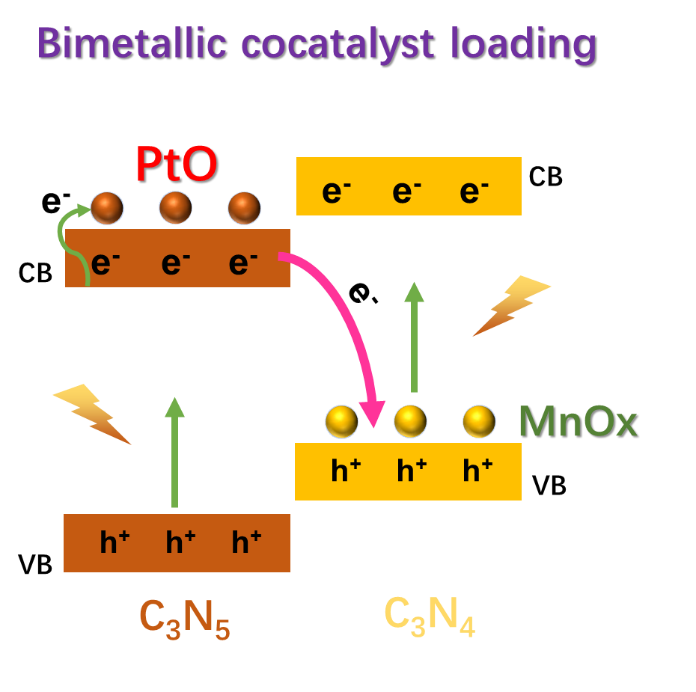


**Figure S21** Mechanism diagram of photogenerated electron transfer at the interface of CNHJ after bimetallic cocatalyst loading.


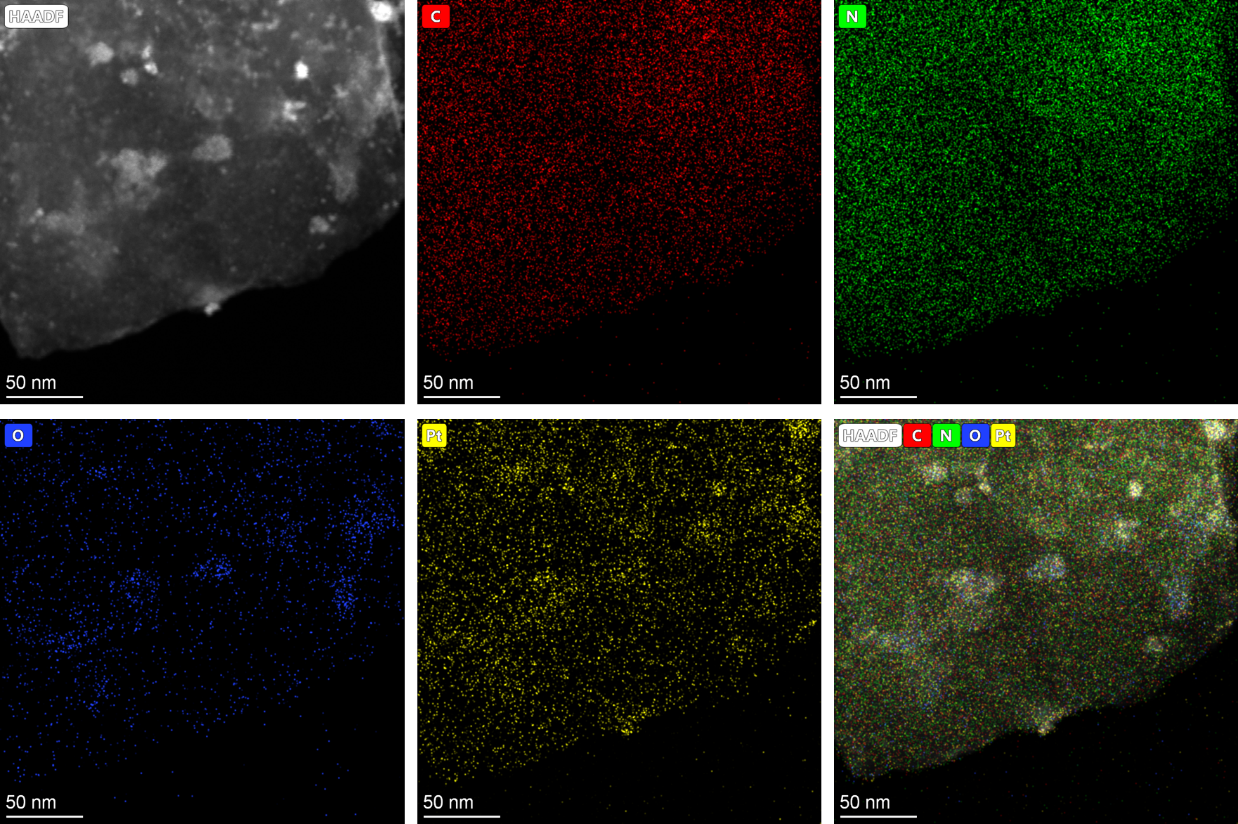


**Figure S22** HAADF-STEM image and corresponding elemental mapping pictures of C_3_N_5_@Pt.

**
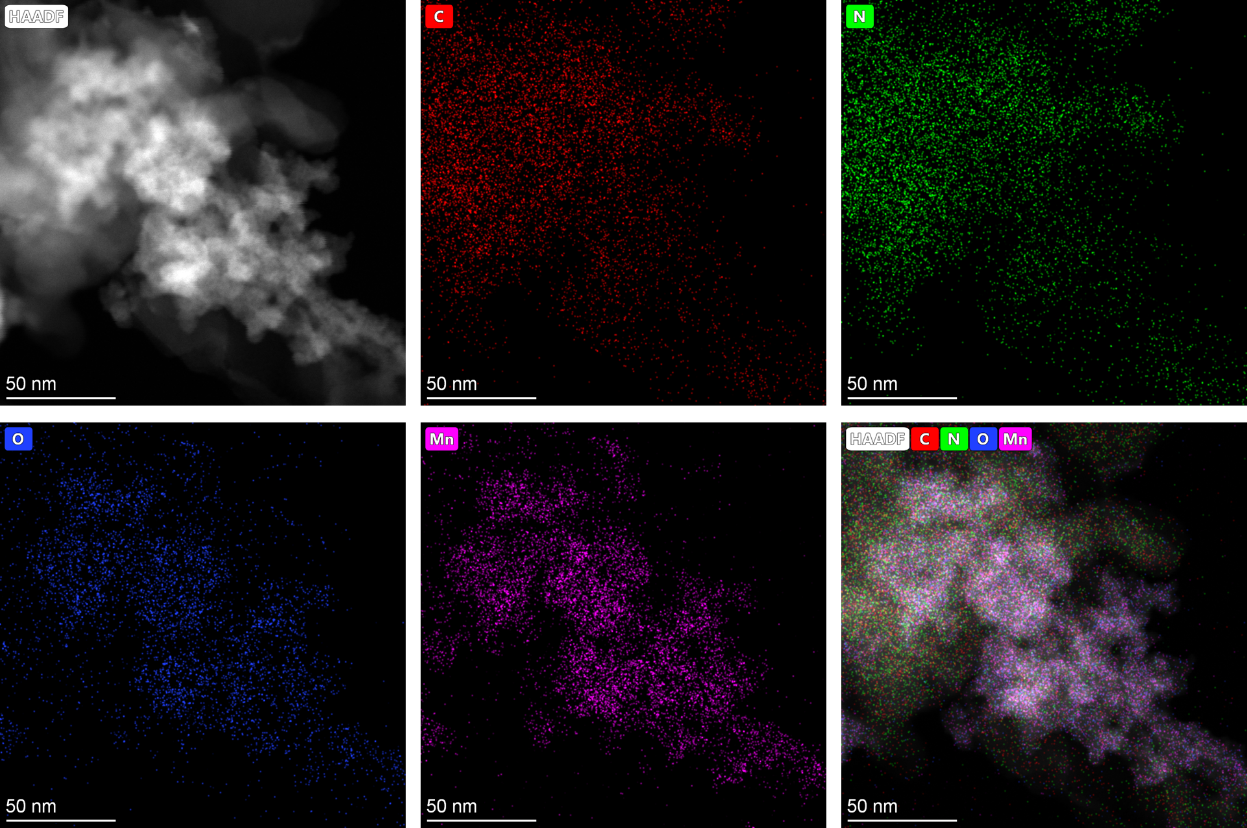
**

**Figure S23** HAADF-STEM image and corresponding elemental mapping pictures of C_3_N_4_@Mn.


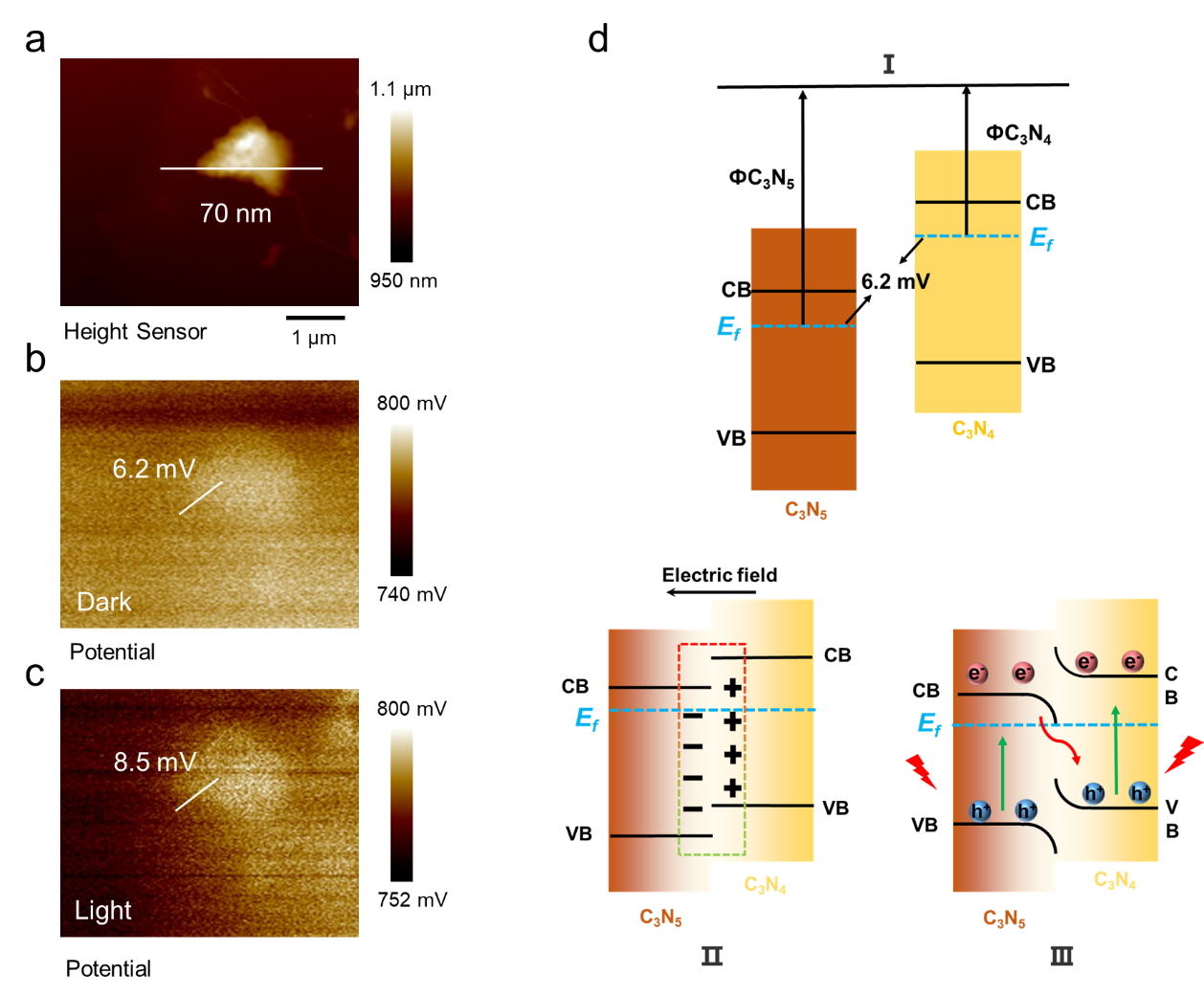


**Figure S24** (a) AFM image of CNHJ. (b) The distribution of potential difference at the interface of CNHJ in a dark environment or (c) under illumination. (d) Schematic of the S-scheme migration of photogenerated carriers induced by the electric field at the CNHJ interface. Processes I and II represent the formation of the built-in electric field owing to the difference in the work function, and process III represents the S-scheme charge separation.


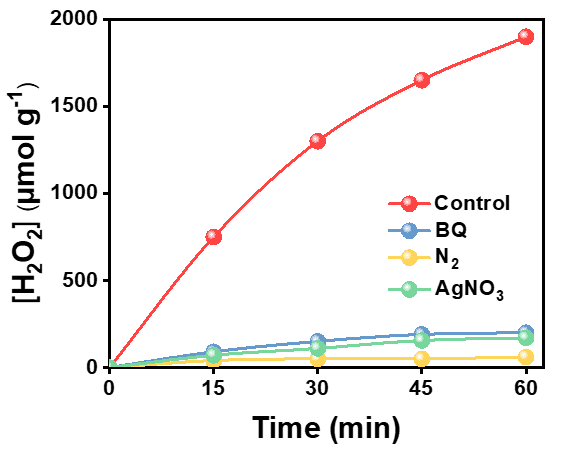


**Figure S25** Photocatalytic H_2_O_2_ yield of CNHJ in the presence of different saturated gases and scavengers.

**Figure S26** In-situ EPR spectra of CNHJ under dark and visible light conditions.


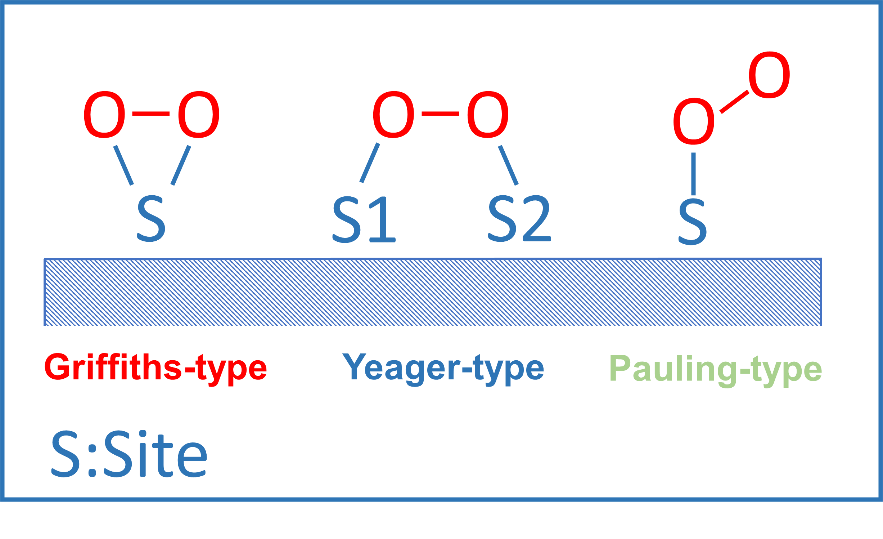


**Figure S27** Schematic diagram of O_2_ adsorption models in reaction sites in H_2_O_2_ photosynthesis.


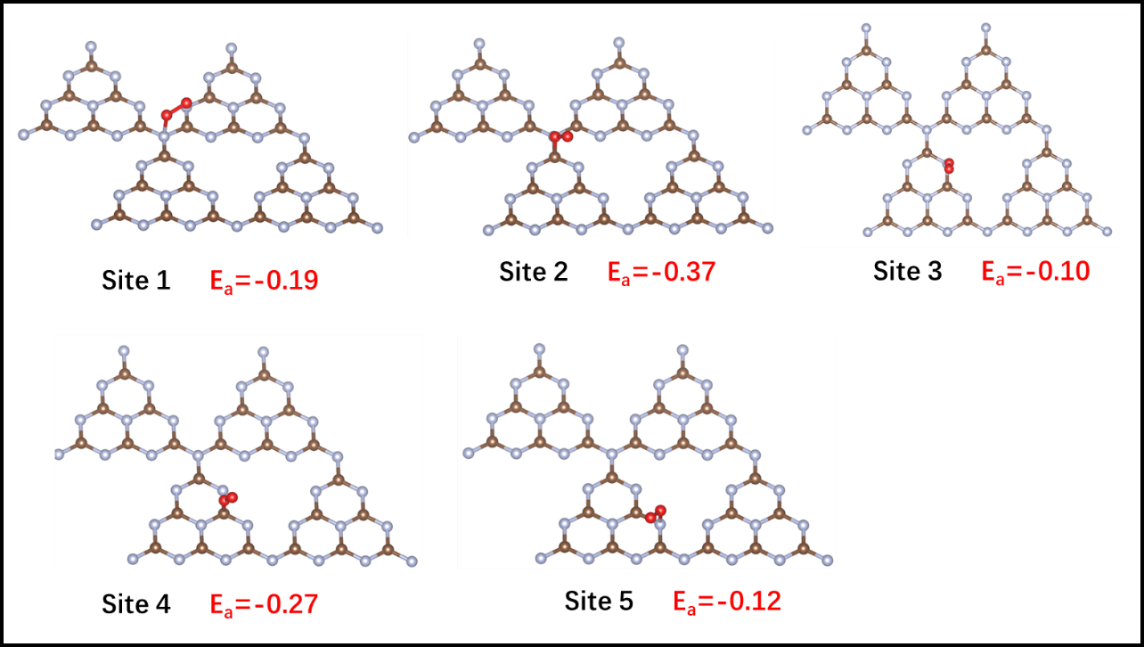


**Figure S28** Optimal models and sites for O_2_ adsorption on C_3_N_4_.


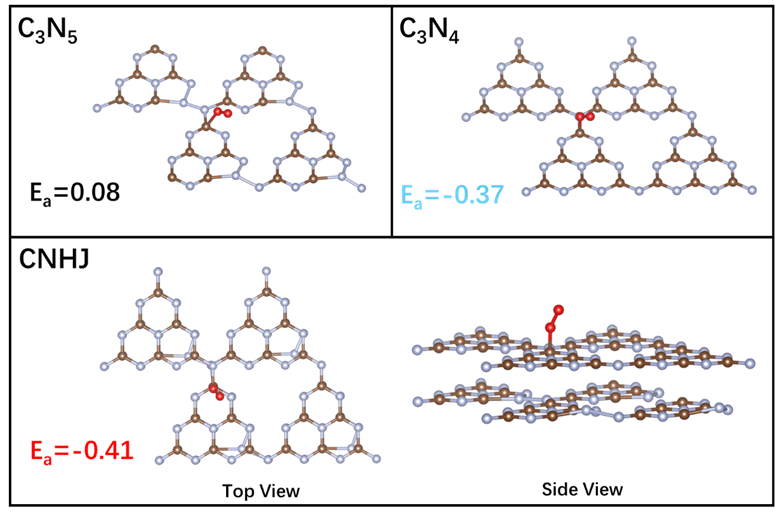


**Figure S29** Calculated models and adsorption energies for O_2_ adsorption on C_3_N_5_, C_3_N_4_ and CNHJ.

**Figure S30** The O_2_-TPD signals of C_3_N_5_, C_3_N_4_ and CNHJ.

**Figure S31** In situ DRIFT spectrum analysis over CNHJ.

**Table S1** Total energy (eV) of the C_3_N_4_, C_3_N_5_ and CNHJ layers and corresponding intermediate in O_2_ reduction reaction.

|  | O_2_ | *O_2_ | *OOH | *H_2_O_2_ | H_2_O_2_ |
| --- | --- | --- | --- | --- | --- |
| C_3_N_4_ | 0 | -0.37 eV | -0.33 eV | -0.79 eV | -1.02 eV |
| C_3_N_5_ | 0 | 0.08 eV | -0.21 eV | -0.62 eV | -1.02 eV |
| CNHJ | 0 | -0.41 eV | -0.59 eV | -0.96 eV | -1.02 eV |
